# A facile access and evaluation of some novel thiazole and 1,3,4-thiadiazole derivatives incorporating thiazole moiety as potent anticancer agents

**DOI:** 10.1186/s13065-017-0335-8

**Published:** 2017-10-16

**Authors:** Sobhi M. Gomha, Mohamad R. Abdelaziz, Nabila A. Kheder, Hassan M. Abdel-aziz, Seham Alterary, Yahia N. Mabkhot

**Affiliations:** 1 Department of Chemistry, Faculty of Science, Cairo University, Giza, 12613 Egypt; 2 Department of Pharmaceutical Chemistry, Faculty of Pharmacy, MIU University, Cairo, Egypt; 3 Department of Pharmaceutical Chemistry, Faculty of Pharmacy, King Khalid University, Abha, 61441 Saudi Arabia; 4 Department of Chemistry, Faculty of Science, University of Beni Suef, Beni Suef, Egypt; 5 Department of Chemistry, College of Science, King Saud University, P. O. Box 2455, Riyadh, 11451 Saudi Arabia

**Keywords:** Thiazoles, Thiadiazoles, Hydrazonoyl chlorides, Phenacyl bromide, Thioamide, Anticancer activity

## Abstract

**Background:**

Many heterocyclic compounds containing thiazole or 1,3,4-thiadiazole ring in their skeletons have been reported to possess various pharmacological activities especially anticancer activities.

**Results:**

4-Methyl-2-phenylthiazole-5-carbohydrazide (**2**) was used as a synthon to prepare 2-(4-methyl-2-phenylthiazole-5-carbonyl)-*N*-phenylhydrazinecarbothioamide (**3**) and 2-(2-(4-methyl-2-phenylthiazole-5-carbonyl)hydrazono)-*N*′-phenylpropane hydrazonoyl chlorides **5a**–**c**. In addition, thioamide **3** was used as starting material for preparation of a new series of thiadiazole derivatives via its reaction with hydrazonoyl chlorides **5a**–**c** in dioxane using triethylamines as catalyst. In addition, a series of thiazole derivatives was synthesized by reaction of thioamide **3** with a number of α-halo compounds, namely, 3-chloropentane-2,4-dione (**8**) or 2-chloro-3-oxo-*N*-phenyl butanamide (**10**) phenacyl bromide **12** ethyl chloroacetate (**14**) in EtOH in the presence of triethylamine. The structures assigned for all the new products were elucidated based on both elemental analyses and spectral data and the mechanisms of their formation was also discussed. Moreover, the new products was evaluated in vitro by MTT assays for their anticancer activity against cell lines of Hepatocellular carcinoma cell line (HepG-2). The best result observed for compounds **7b** (IC_50_ = 1.61 ± 1.92 (μg/mL)) and **11** (IC_50_ = 1.98 ± 1.22 (μg/mL)). The structure–activity relationships have been suggested based on their anticancer results.

**Conclusions:**

A novel series of new pharmacophores containing thiazole moiety have been synthesized using a facile and convenient methods and evaluated as potent anticancer agents.
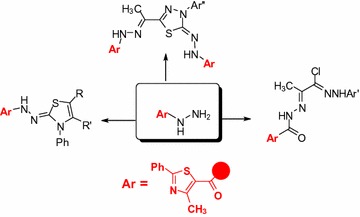

## Introduction

**Fig. 1:**
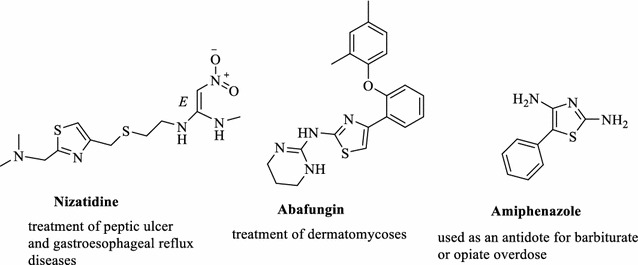
Some marketed drugs containing thiazole ring

Identification of novel structure leads that may be of use in designing new, potent, selective and less toxic anticancer agents remains a major challenge for medicinal chemistry researchers. Compounds containing thiazole core have diverse biological activities as antihypertension, antifungal, antimicrobial, anti-inflammatory, antioxidant, antitubercular [1–7], and anticancer [8–12]. Also, thiazole ring present in many drugs such as Nizatidine, Abafungin, and Amiphenazole (Fig. 1).

**Fig. 2:**
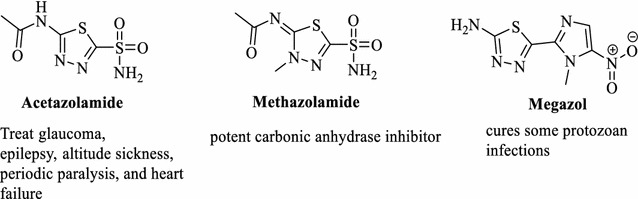
Examples of drugs containing a 1,3,4-thiadiazole ring

Many biological activities were reported for the compounds containing 1,3,4-thiadiazole ring such as antituberculosis, anti-inflammatory, antidepressant and anxiolytic, antioxidant, anticonvulsants [13–17] and anticancer activities [18–20]. In addition, many drugs containing 1,3,4-thiadiazole ring are available in the market such as acetazolamide, methazolamide, and megazol (Fig. 2).

In continuation of our studies dealing with the utility of hydrazonoyl halides for synthesis of various bioactive bridgehead nitrogen polyheterocycles [21–30], we wish to report herein a new facile synthesis of new heterocycles containing thiazole and 1,3,4-thiadiazole or two thiazole rings in one molecular frame. We anticipated that the synthesized compounds would have potent pharmacological activities.

## Results and discussion

### Chemistry

**Scheme 1:**
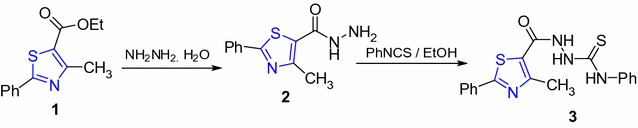
Synthesis of thiazoles **2**,**3**

2-(4-Methyl-2-phenylthiazole-5-carbonyl)-*N*-phenylhydrazinecarbothioamide (**3**) [31] was prepared via reaction of 4-methyl-2-phenylthiazole-5-carbohydrazide (**2**) with phenyl isothiocyanate in EtOH (Scheme 1).

**Scheme 2:**
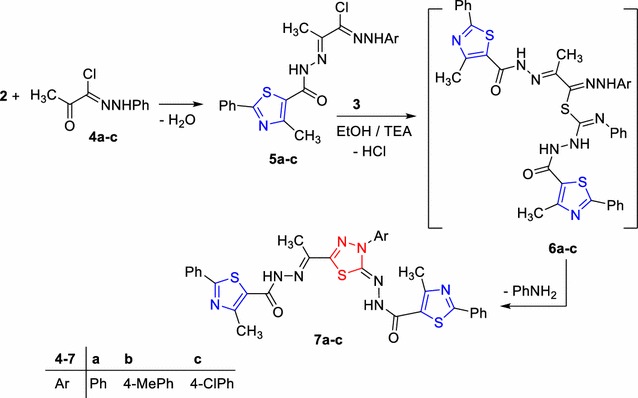
Synthesis of thiadiazole derivatives **7a**–**c**

The reaction of compound **2** with the appropriate hydrazonoyl chlorides **4a**–**c** [32] in refluxing ethanol yielded the corresponding condensation product **5** (Scheme 2). The IR spectra of the latter products revealed a carbonyl and two NH absorption bands (see “Experimental” part). Their ^1^HNMR showed two D_2_O exchangeable signals of two NH protons in the regions δ 10.03–10.06 and δ 10.57–10.59 ppm. Also, their mass spectra confirmed the assigned structure **5** (Scheme 2). Treatment of thioamide derivative **3** with the appropriate hydrazonoyl halides of type **5a**–**c** in refluxing EtOH containing TEA gave the corresponding thiadiazole derivatives **7a**–**c** (Scheme 2). Their structures were elucidated on the basis of their spectral data and elemental analysis (see “Experimental”).

**Scheme 3:**
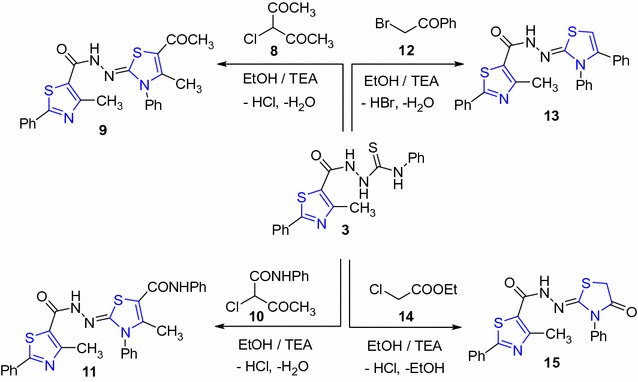
Synthesis of thiazole derivatives **9**, **11**, **13** and **15**

Next, refluxing of compound **3** with 3-chloropentane-2,4-dione (**8**) or 2-chloro-3-oxo-*N*-phenyl butanamide (**10**) in EtOH in the presence of triethylamine afforded the thiazole derivatives **9** and **11**, respectively (Scheme 3).The structure of compounds **9** and **11** were elucidated based on their elemental analysis and spectral data (see “Experimental”).

In a similar manner, thioamide **3** reacted with phenacyl bromide **12** under the same experimental condition to afford one isolable product **13** named as *N*′-(3,4-diphenylthiazol- 2(3H)-ylidene)-4-methyl-2-phenyl thiazole-5-carbohydrazide (Scheme 3). The structure of thiazole **13** was established based on its elemental analysis and spectral data (see “Experimental”).

Finally, thioamide derivative **3** reacted with ethyl chloroacetate (**14**) to afford thiazole **15** as showed in Scheme 3. Its IR spectrum showed absorption bands at v 3331 (NH), and 1726, 1648 (2C=O) cm^−1^. In addition, its ^1^HNMR spectrum showed singlet signal at δ 4.23 ppm due to the thiazolidinone (CH_2_) group.

### Anticancer activity

**Fig. 3:**
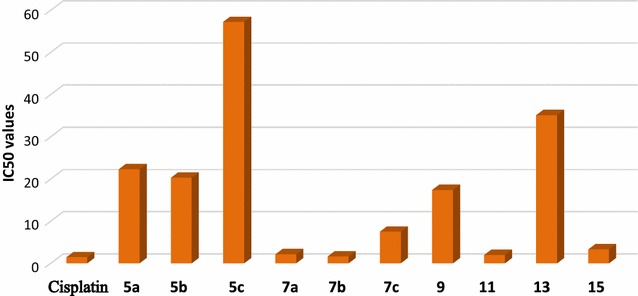
Comparison of the IC_50_ of the new synthesized compounds against Cisplatin

The synthesized compounds were tested as anticancer agents against human Hepatocellular carcinoma cell line (HepG-2) using colorimetric MTT assay. We also included the well-known anticancer standard drug (Cisplatin) in the same assay to compare the potency of the synthesized compounds. The IC_50_ (the concentration of test compounds required to kill 50% of cell population) was determined (Table 1, Fig. 3).

**Table 1:** The in vitro inhibitory activity of the tested compounds against tumor cell lines expressed as IC_50_ values (μg/mL) ± standard deviation from three replicates

Tested compounds	IC_50_ (μg/mL)	Tested compounds	IC_50_ (μg/mL)
Cisplatin	1.43 ± 2.03	**7c**	7.51 ± 0.64
**5a**	22.3 ± 2.41	**9**	17.4 ± 0.73
**5b**	20.3 ± 3.70	**11**	1.98 ± 1.22
**5c**	57.2 ± 7.12	**13**	35.1 ± 10.8
**7a**	2.14 ± 3.54	**15**	3.31 ± 2.65
**7b**	1.61 ± 1.92

**Fig. 4:**
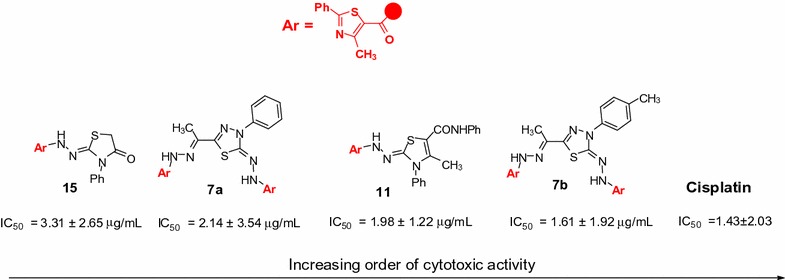
The ascending order of the cytotoxic activity

The results of Table 1 revealed that the ascending order of the cytotoxic activity of the newly synthesized compounds towards the human Hepatocellular carcinoma cell line (HepG-2) were as follow: **5c** < **13** < **5a** < **5b** < **9** < **7c** < **15** < **7a** < **11** < **7b** (Fig. 4).

From the data of Table 1, we concluded the following structure–activity relationships (SARs):

- The thiazole ring is essential for the activity.
- Less number of thiazole ring as in compounds **5a**–**c** lead to drastic drop in activity.
- 1,3,4-Thiadiazole ring is crucial for the cytotoxic activity.
- Presence of methyl group (electron donating group) at position 4 of the phenyl ring in compound **7b** increase its activity more than compound **7a**.
- The presence of the N-phenylcarboxamide group in compound **11** leads to increasing of its cytotoxic activity.

## Experimental

### Chemistry

#### General

Melting points were measured on an Electrothermal IA 9000 series digital melting point apparatus (Bibby Sci. Lim. Stone, Staffordshire, UK). IR spectra were measured on PyeUnicamSP 3300 and Shimadzu FTIR 8101 PC infrared spectrophotometers (Shimadzu, Tokyo, Japan) in potassium bromide discs. NMR spectra were measured on a Varian Mercury VX-300 NMR spectrometer (Varian, Inc., Karlsruhe, Germany) operating at 300 MHz (^1^HNMR) and run in deuterated dimethylsulfoxide (DMSO-*d*_*6*_). Chemical shifts were related to that of the solvent. Mass spectra were recorded on a Shimadzu GCMS-QP1000 EX mass spectrometer (Tokyo, Japan) at 70 eV. Elemental analyses were measured by using a German made Elementarvario LIII CHNS analyzer. 2-(4-Methyl-2-phenylthiazole-5-carbonyl)-*N*-phenylhydrazinecarbothioamide (**3**) [31], and hydrazonoyl halides **4a**–**c** [32] were prepared as reported in the respective literature.

### Synthetic procedures

#### Synthesis of hydrazonoyl chlorides 5a–c

A mixture of 4-methyl-2-phenylthiazole-5-carbohydrazide (**2**) (2.33 g, 10 mmol) and the appropriate hydrazonoyl chlorides **4a**–**c** (10 mmol) in ethanol (30 mL) was refluxed for 3–5 h (monitored through TLC).The resulting solid product was collected and recrystallized from the proper solvent to give the corresponding products **5a**–**c**.

##### 2-(2-(4-Methyl-2-phenylthiazole-5-carbonyl)hydrazono)-*N*′-phenylpropane hydrazonoyl chloride (5a)

Yellow solid; yield (84%); m.p. 188–190 °C (EtOH); IR (KBr) v 3440, 3316 (2NH), 3036, 2922 (CH), 1640 (C=O), 1599 (C=N) cm^−1^; ^1^H NMR (DMSO-*d*_*6*_) δ 2.36 (s, 3H, CH_3_), 2.76 (s, 3H, CH_3_), 7.06–7.86 (m, 10H, ArH), 10.03 (s, br, 1H, D_2_O-exchangeable NH), 10.57 (s, br, 1H, D_2_O-exchangeable NH); MS m/z (%): 413 (M^+^+2, 12), 411 (M^+^, 40), 375 (48), 202 (100), 174 (45), 71 (26). Anal. Calcd for C_20_H_18_ClN_5_OS (411.91): C, 58.32; H, 4.40; N, 17.00. Found: C, 58.19; H, 4.37; N, 16.88%.

##### 2-(2-(4-Methyl-2-phenylthiazole-5-carbonyl)hydrazono)-N′-(p-tolyl)propane- hydrazonoylchloride (5b)

Yellow solid; yield (86%); m.p. 172–174 °C (EtOH); IR (KBr) v 3437, 3313 (2NH), 3041, 2917 (CH), 1679 (C=O), 1598 (C=N) cm^−1^; ^1^H NMR (DMSO-*d*_*6*_) δ 2.24 (s, 3H, CH_3_), 2.34 (s, 3H, CH_3_), 2.77 (s, 3H, CH_3_), 7.08–7.99 (m, 9H, ArH), 10.06 (s, br, 1H, D_2_O-exchangeable NH), 10.59 (s, br, 1H, D_2_O-exchangeable NH); MS m/z (%) 427 (M^+^+2, 10), 425 (M^+^, 33), 389 (26), 202 (81), 106 (100), 64 (66). Anal. Calcd for C_21_H_20_ClN_5_OS (425.93): C, 59.22; H, 4.73; N, 16.44. Found: C, 59.18; H, 4.65; N, 16.37%.

##### N′-(4-Chlorophenyl)-2-(2-(4-methyl-2-phenylthiazole-5-carbonyl)hydrazono) propane hydrazonoyl chloride (5c)

Yellow solid; yield (87%); m.p. 194–196 °C (DMF); IR (KBr) v 3434, 3319 (2NH), 3044, 2926 (CH), 1682 (C=O), 1593 (C=N) cm^−1^; ^1^H NMR (DMSO-*d*_*6*_) δ 2.37 (s, 3H, CH_3_), 2.77 (s, 3H, CH_3_), 7.08–7.99 (m, 9H, Ar–H), 10.06 (s, br, 1H, D_2_O-exchangeable NH), 10.57 (s, br, 1H, D_2_O-exchangeable NH); MS m/z (%) 446 (M^+^, 8), 283 (14), 202 (39), 104 (46), 80 (100), 64 (90). Anal. Calcd for C_20_H_17_Cl_2_N_5_OS (446.35): C, 53.82; H, 3.84; N, 15.69. Found: C, 53.75; H, 3.79; N, 15.58%.

#### Synthesis of 1,3,4-thiadiazole derivatives 7a–c

A mixture of compound **3** (0.368 g, 1 mmol) and the appropriate hydrazonoyl chlorides **5a**–**c** (1 mmol) in ethanol (20 mL) containing triethylamine (0.1 g, 1 mmol) was refluxed for 6 h. The formed solid product was filtered, washed with methanol, dried and recrystallized from the suitable solvents to give corresponding products **7a**–**c**.

##### 4-Methyl-N′-(1-(-5-(2-(4-methyl-2-phenylthiazole-5-carbonyl)hydrazono)-4-phenyl-4,5-dihydro-1,3,4-thiadiazol-2-yl)ethylidene)-2-phenylthiazole-5-carbohydrazide(7a)

Yellow solid; yield (74%); m.p. 162–164 °C (EtOH); IR (KBr) v 3421, 3307 (2NH), 3031, 2951 (CH), 1649 (C=O), 1596 (C=N) cm^−1^; ^1^H NMR (DMSO-*d*_*6*_) δ 2.34 (s, 3H, CH_3_), 2.66 (s, 3H, CH_3_), 2.76(s, 3H, CH_3_), 6.97-8.14 (m, 15H, ArH), 10.18 (s, br, 1H, D_2_O-exchangeable NH), 11.17 (s, br, 1H, D_2_O-exchangeable NH); MS m/z (%) 650 (M^+^, 34), 526 (30), 416 (60), 358 (28), 104 (55), 64 (100). Anal. Calcd for C_32_H_26_N_8_O_2_S_3_ (650.80): C, 59.06; H, 4.03; N, 17.22. Found C, 58.94; H, 4.01; N, 17.07%.

##### 4-Methyl-N′-(1-(5-(2-(4-methyl-2-phenylthiazole-5-carbonyl)hydrazono)-4-(p-tolyl)-4,5-dihydro-1,3,4-thiadiazol-2-yl)ethylidene)-2-phenylthiazole-5-carbohydrazide (7b)

Yellow solid; yield (72%); m.p. 149–151 °C (EtOH); IR (KBr) v 3422, 3328 (2NH), 3053, 2929 (CH), 1647 (C=O), 1597 (C=N) cm^−1^; ^1^H NMR (DMSO-*d*_*6*_) δ 2.26 (s, 3H, CH_3_),2.35 (s, 3H, CH_3_), 2.65 (s, 3H, CH_3_), 2.76(s, 3H, CH_3_), 6.91–8.03 (m, 14H, ArH), 10.18 (s, br, 1H, D_2_O-exchangeable NH), 11.14 (s, br, 1H, D_2_O-exchangeable NH); MS m/z (%) 664 (M^+^, 35), 553 (60), 334 (19), 202 (65), 104 (85), 64 (100). Anal. Calcd for C_33_H_28_N_8_O_2_S_3_ (664.82): C, 59.62; H, 4.25; N, 16.85. Found C, 59.47; H, 4.17; N, 16.79%.

##### N′-(3-(4-Chlorophenyl)-5-(1-(2-(4-methyl-2-phenylthiazole-5-carbonyl)hydrazono)eth-yl)-1,3,4-thiadiazol-2(3H)-ylidene)-4-methyl-2-phenylthiazole-5-carbohydrazide (7c)

Yellow solid; yield (76%); m.p. 191–193 °C (Dioxane); IR (KBr) v 3424, 3312 (2NH), 3047, 2932 (CH), 1649 (C=O), 1599 (C=N) cm^−1^; ^1^H NMR (DMSO-*d*_*6*_) δ 2.33 (s, 3H, CH_3_), 2.66 (s, 3H, CH_3_), 2.77(s, 3H, CH_3_), 6.90–8.11 (m, 14H, ArH), 10.13 (s, br, 1H, D_2_O-exchangeable NH), 11.19 (s, br, 1H, D_2_O-exchangeable NH); MS m/z (%) 686 (M^+^+2, 8), 684 (M+, 26), 513 (53), 368 (39), 257 (17), 104 (25), 64 (100). Anal.Calcd for C_32_H_25_ClN_8_O_2_S_3_ (685.24): C, 56.09; H, 3.68; N, 16.35. Found C, 56.02; H, 3.58; N, 16.22%.

#### General procedure for the synthesis of thiazole derivatives 9, 11, 13, and 15

A mixture of compound **3** (0.368 g, 1 mmol) and the appropriate α-halo-compounds namely, 3-chloropentane-2,4-dione (**8**), 2-chloro-3-oxo-N-phenylbutanamide (**10**), 2-bromo-1-phenyl ethanone (**12**) and ethyl 2-chloroacetate (**14**) (1 mmol for each) in ethanol (20 mL) containing triethylamine (0.1 g, 1 mmol) was refluxed for 4–6 h. (monitored by TLC The solid product was filtered, washed with water, dried and recrystallized from the proper solvent to give the corresponding thiazole derivatives **9**, **11**, **13**, and **15**, respectively.

##### N′-(5-Acetyl-4-methyl-3-phenylthiazol-2(3H)-ylidene)-4-methyl-2-phenylthiazole-5-carbohydrazide (9)

Yellow solid; yield (78%); m.p. 155–157 °C (EtOH); IR (KBr) v 3432 (NH), 3036, 2993 (CH), 1695, 1648 (2C=O), 1590 (C=N) cm^−1^; ^1^H NMR(DMSO-*d*_*6*_) δ 2.32 (s, 3H, CH_3_),2.46 (s, 3H, CH_3_), 2.77 (s, 3H, CH_3_), 6.91–7.86 (m, 10H, ArH), 10.61 (s, br, 1H, D_2_O-exchangeable NH); MS m/z (%) 448 (M^+^, 57), 246 (60), 176 (35), 104 (80), 77 (100). Anal.Calcd for C_23_H_20_N_4_O_2_S_2_ (448.56): C, 61.59; H, 4.49; N, 12.49. Found C, 61.48; H, 4.36; N, 12.37%.

##### 4-Methyl-2-(2-(4-methyl-2-phenylthiazole-5-carbonyl)hydrazono)-*N*-3-diphenyl-2,3-dihydrothiazole-5-carboxamide (11)

Yellow solid; yield (79%); m.p. 182–84 °C (DMF); IR (KBr): v 3435, 3176 (2NH), 3030, 2928(CH), 1671, 1649 (2C=O), 1594 (C=N) cm^−1^; ^1^H NMR (DMSO-*d*_*6*_) δ 2.36 (s, 3H, CH_3_),2.76(s, 3H, CH_3_), 6.97–7.73 (m, 15H, ArH), 10.46 (s, br, 1H, D_2_O-exchangeable NH), 11.72 (s, br, 1H, D_2_O-exchangeable NH); MS m/z (%) 525 (M^+^, 7), 447 (16), 334 (100), 200 (59), 77 (89). Anal.Calcd for C_28_H_23_N_5_O_2_S_2_ (525.64): C, 63.98; H, 4.41; N, 13.32. Found C, 63.84; H, 4.30; N, 13.28%.

##### N′-(3,4-Diphenylthiazol-2(3H)-ylidene)-4-methyl-2-phenylthiazole-5-carbohydrazide (13)

Yellow solid; yield (70%); m.p. 174–178 °C (EtOH); IR (KBr) v 3369 (NH), 3047, 2926(CH), 1648 (C=O), 1594 (C=N) cm^−1^; ^1^H NMR (DMSO-*d*_*6*_) δ 2.75 (s, 3H, CH_3_), 7.03 (s, 1H, thiazole-H5), 7.35–8.02 (m, 15H, ArH), 10.73 (s, br, 1H, D_2_O-exchangeable NH); MS m/z (%) 468 (M^+^, 25), 334 (100), 200 (40), 104 (69), 64(65). Anal.Calcd for C_26_H_20_N_4_OS_2_ (468.59): C, 66.64; H, 4.30; N, 11.96. Found C, 66.55; H, 4.21; N, 11.79%.

##### 4-Methyl-N′-(4-oxo-3-phenylthiazolidin-2-ylidene)-2-phenylthiazole-5-carbo- hydrazide (15)

Yellowish-white solid; yield (72%); m.p. 192–194 °C (Dioxane); IR (KBr) v 3331(NH), 3036, 2926 (CH), 1726, 1648 (2C=O), 1596 (C=N) cm^−1^; ^1^H NMR (DMSO-*d*_*6*_) δ 2.65 (s, 3H, CH_3_), 4.23 (s, 2H, thiazolone-CH_2_), 7.40–7.88 (m, 10H, ArH), 10.82 (s, br, 1H, D_2_O-exchangeable NH); MS m/z (%) 408 (M^+^, 65), 334 (18), 202 (100), 104 (86), 64 (69). Anal.Calcd for C_20_H_16_N_4_O_2_S_2_ (408.50): C, 58.80; H, 3.95; N, 13.72. Found C, 58.68; H, 3.84; N, 13.64%.

### Anticancer activity

The cytotoxic evaluation of the synthesized compounds was carried out at the Regional Center for Mycology and Biotechnology at Al-Azhar University, Cairo, Egypt according to the reported method [33].

## Conclusions

We successfully synthesized a series of novel heterocycles containing thiazole and 1,3,4-thiadiazole rings by a facile and convenient method. The structure of the newly prepared compounds was established based on both elemental analysis and spectroscopic data. The anticancer activity of the synthesized compounds was measured and showed promising activity.

## Abbreviations

HepG2: human hepatocellular carcinoma
EtOH: ethanol
m.p.: melting point
TEA: triethylamine
IR: infra-red
ATCC: American Type Culture Collection
TLC: thin layer chromatography
